# Animal Models of Diabetes and Its Associated Complications

**DOI:** 10.1155/2013/593204

**Published:** 2013-10-28

**Authors:** Md. Shahidul Islam, Daisuke Koya, Bernard Portha

**Affiliations:** ^1^Department of Biochemistry, School of Life Sciences, University of KwaZulu-Natal (Westville Campus), Durban 4000, South Africa; ^2^Department of Diabetology & Endocrinology, Kanazawa Medical University, Kahoku-gun, Ishikawa 920-0293, Japan; ^3^Laboratory of Biology and Pathology of the Endocrine Pancreas, University of Paris-Diderot and CNRS, 75013 Paris, France

Diabetes is a major threat to global public health. According to the latest data from the International Diabetes Federation (IDF), at least 366 million people are living with diabetes and this number is projected to be 552 million by 2030. At least 50% of people with diabetes suffer from one or two major diabetic complications such as diabetic cardiomyopathy, nephropathy, neuropathy, retinopathy, and diabetic foot diseases. Development of an authentic model of each type of diabetes and their associated complications, which exactly mimic the human pathogenesis, is very crucial not only for a better understanding of the core causes of the diseases but also for the development and routine pharmacological screening of novel anti-diabetic drugs. A number of genetic and nongenetic animal models of the different types of diabetes and their associated complications have been developed in last three decades but none of them are without limitations.

This special issue of the Journal of Diabetes Research has been designed to publish original and review articles with novel approaches of the development of animal model of diabetes and its associated complications. Almost all areas of the animal models of diabetes and its associated complications have been covered by this issue. Among the reviews: the animal models of diabetic retinopathy by A. K. W. Lai and A. Lo, animal models of diabetic neuropathy by M. S. Islam, murine models of diabetic neuropathy by L. L. Kong et al., methods and models for metabolic assessment by G. Pacini et al., and porcine models of accelerated coronary atherosclerosis by D. Hamamdzic et al. contributed significantly by summarizing the advantages, disadvantages, and suitability of animal models developed to study the above-mentioned diabetic complications. Many other original papers such as role of adrenomedulin in the renal NADPH oxidase and (pro)renin in diabetic mice by M. Hayashi et al., development of glomerulopathy in the KK.Cg-Ay/J mouse with the pathology of diabetic nephropathy by P. Stephen et al., and the role of ERK/MAPK and TGF-beta/Smad signaling pathways in the kidney fibrosis of diabetic mice by X. Cheng et al. contributed novel information in the pathogenesis of diabetic nephropathy, a major and common complications of diabetes mellitus. Finally, an exceptional paper has been contributed by E. Hillhouse et al. on the effect of nearby construction on the progress to overt autoimmune diabetes in NOD mice. As a whole, papers from almost every aspects of the animals models of diabetes and its associated complications have been published in this special issue so it can be a special interest of diabetes researchers particularly those who are working in the different areas of diabetic complications.

*Md.  Shahidul  Islam* *Md.  Shahidul  Islam* *Daisuke  Koya* *Daisuke  Koya* *Bernard  Portha* *Bernard  Portha*

